# A case of a tiny neuroendocrine carcinoma in a large flat-elevated rectal tumor causing multiple liver metastases

**DOI:** 10.1007/s12328-022-01691-9

**Published:** 2022-08-31

**Authors:** Daisuke Ohki, Yosuke Tsuji, Mariko Tanaka, Tetsuo Ushiku, Mitsuhiro Fujishiro

**Affiliations:** 1 Department of Gastroenterology, Graduate School of Medicine, The University of Tokyo, 7-3-1 Hongo, Bunkyo-ku, Tokyo, 113-8655 Japan; 2 Department of Pathology, Graduate School of Medicine, The University of Tokyo, 7-3-1 Hongo, Bunkyo-ku, Tokyo, 113-8655 Japan

**Keywords:** Endoscopic submucosal dissection, Neuroendocrine carcinoma, Neuroendocrine neoplasm, Mixed neuroendocrine–non-neuroendocrine neoplasm, Mixed epithelial endocrine neoplasms

## Abstract

Neuroendocrine tumors are rare malignancies comprising neuroendocrine cells widely distributed in the human body. They occur in various organs of the body, most commonly in the gastrointestinal tract and pancreas in the Japanese population. Mixed neuroendocrine–non-neuroendocrine neoplasm is included in the 2019 WHO classification and defined as having more than 30% of both neuroendocrine and non-neuroendocrine tumor components. However, the number of reports on mixed neuroendocrine and non-neuroendocrine tumors is particularly small. Herein, we encountered a rare case of a tiny neuroendocrine carcinoma in a large flat-elevated rectal tumor resulting in rapid multiple liver metastases. This case was referred to our institution for endoscopic submucosal dissection. Histopathological analysis showed that tubular adenocarcinoma and adenoma were the predominant lesions. Moreover, the neuroendocrine carcinoma component was less than 3% of the total tumor, measuring approximately 5 mm. However, the neuroendocrine carcinoma component was found to be the most advanced part of the infiltrate (T1b at least; SM3.8 mm). Repeat computed tomography 1 week after endoscopic submucosal dissection for evaluating intraoperative perforation revealed liver metastasis, and chemotherapy is currently underway.

## Introduction

The origin of neuroendocrine neoplasms (NENs) can be classified by site into three: foregut (the lung, bronchus, stomach, duodenum, and pancreas); midgut (the small intestine appendix and right half of colon); and hindgut (the left half side of colon). In Japan, NENs are most commonly derived from the pancreas and digestive tract. In the 2019 WHO classification, NENs are classified as neuroendocrine tumor (NET) G1, G2, or G3 and neuroendocrine carcinoma (NEC) according to Ki-67 index and number of fission images [1]. Rectal NEN accounts for 55.7% of gastrointestinal NEN, and is particularly prevalent in the lower rectum [2]. Regarding rectal NETs, tumors less than 1 cm in diameter with a depth of submucosal layers are indicated for endoscopic treatment due to their low lymph node metastasis rate [3]. If histopathological evaluation of resected specimen shows lymphovascular invasion, numerous fission figures, high Ki-67 index, or high grade (G2), the risk of metastasis is high and additional treatment should be considered [4].

On the other hand, gastrointestinal NEC accounts for 6.2% of the gastrointestinal NEN in Japan and, unlike NET G1 G2, has an extremely poor prognosis [2]. Even if NEC has completely resectable local lesion, treatment results of surgery alone are poor. Therefore, resection is recommended as a part of multimodal therapy including drug therapy and radiation therapy. Considering drug therapy, combination therapy including platinum-based drugs is recommended in accordance with treatment of small cell lung cancer [5].

Mixed neuroendocrine–non-NEN (MiNEN) is included in the 2019 WHO classification and defined as having more than 30% of both neuroendocrine and non-NET components. In this classification, tumors previously considered mixed adenoneuroendocrine carcinoma (MANEC) were classified to be included in MiNEN [1].

We describe a case referred to our institution for treatment of a large flat-elevated rectal tumor and underwent endoscopic submucosal dissection (ESD) for treatment diagnosis. The histopathological evaluation of the excised specimen from this case revealed an NEC component in a part of the adenocarcinoma. We report our experience in managing this case.

## Case report

**Fig. 1:**
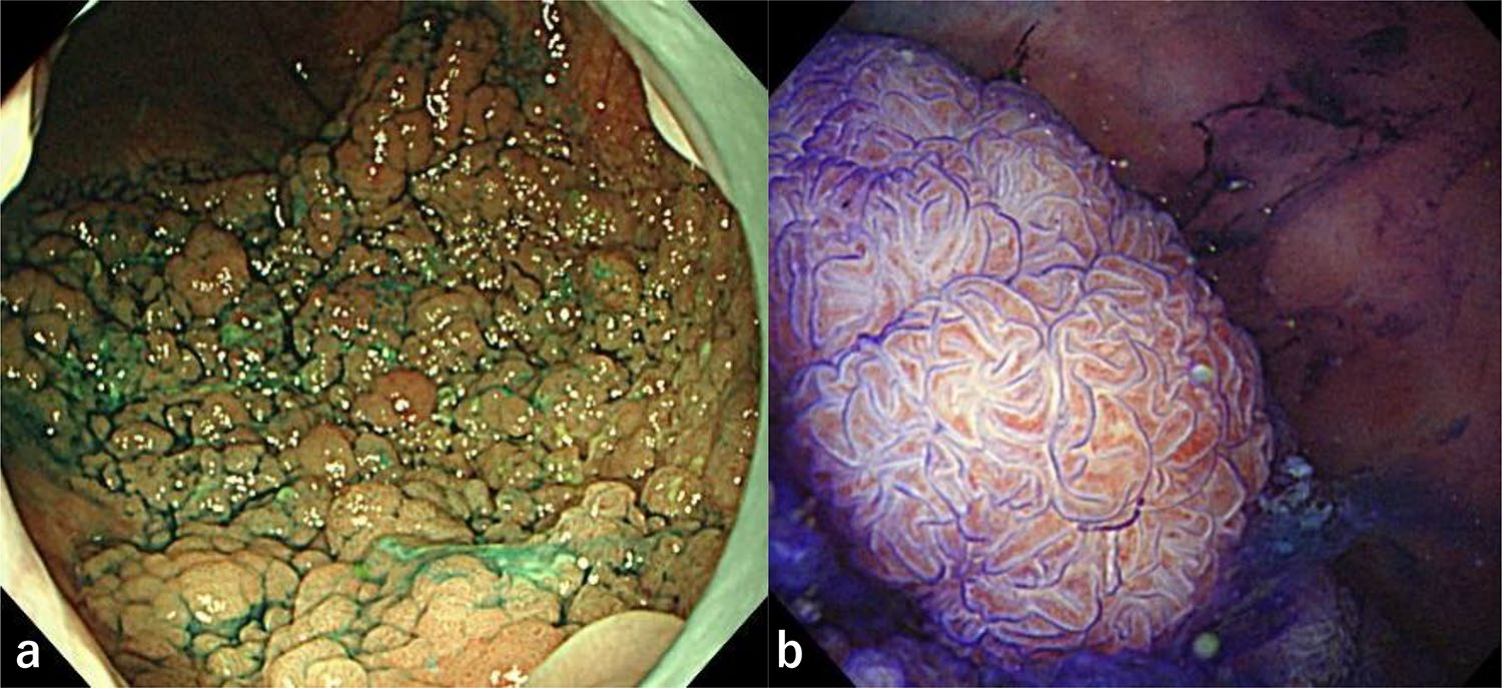
**a** Endoscopic findings of a large flat-elevated rectal tumor. **b** Magnified image after crystal violet staining identified a type IV pit pattern in the lesion

**Fig. 2:**
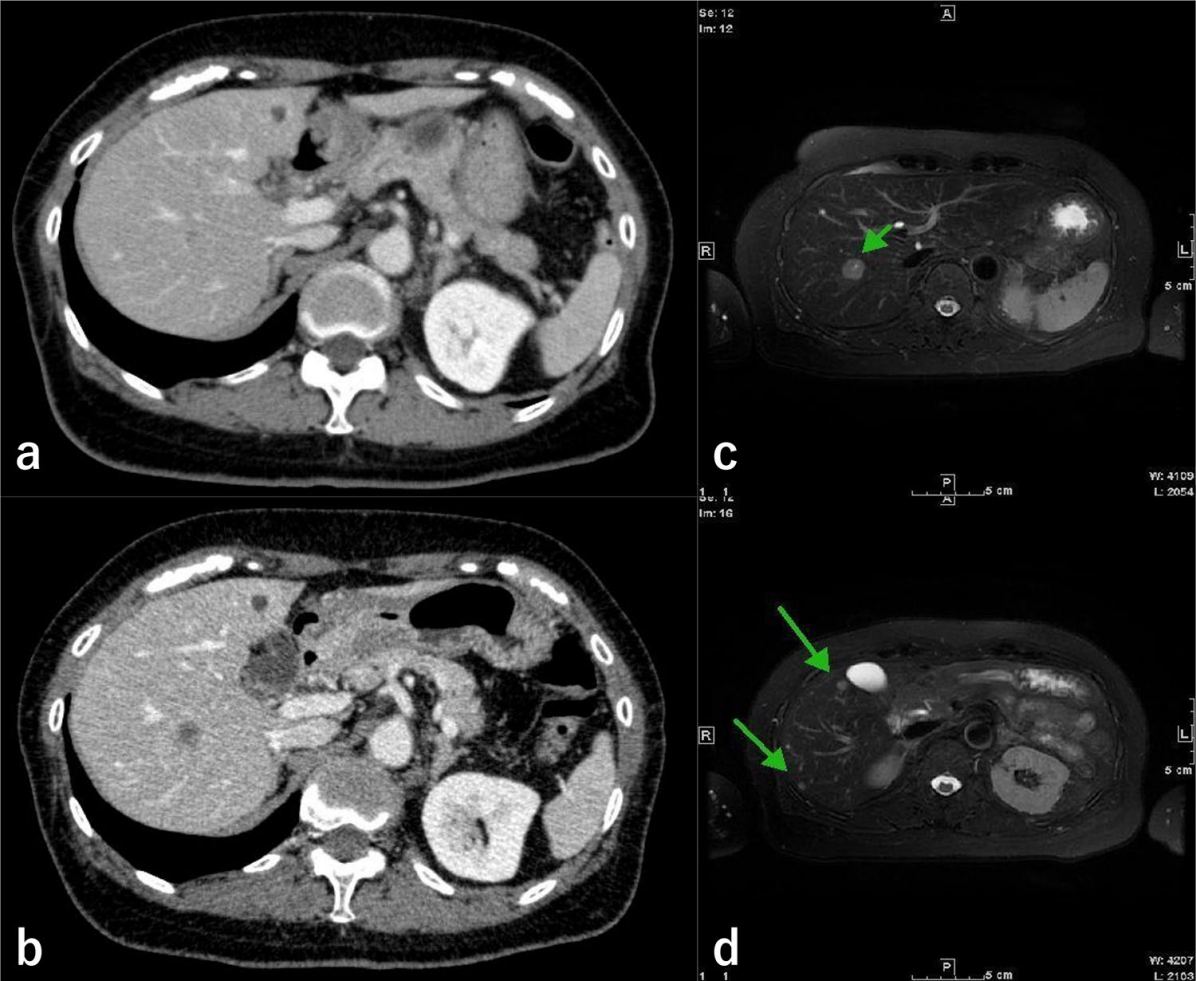
**a** Preoperative CT scan; liver mass is small, pale, indistinct, and unrecognizable. **b** Hepatic mass lesion suspected of hepatic metastasis seen on repeat enhanced CT performed 1 week after endoscopic submucosal dissection (ESD). **c**, **d** Gd-EOB–DTPA–enhanced magnetic resonance imaging (MRI) shows multiple liver metastases

**Fig. 3:**
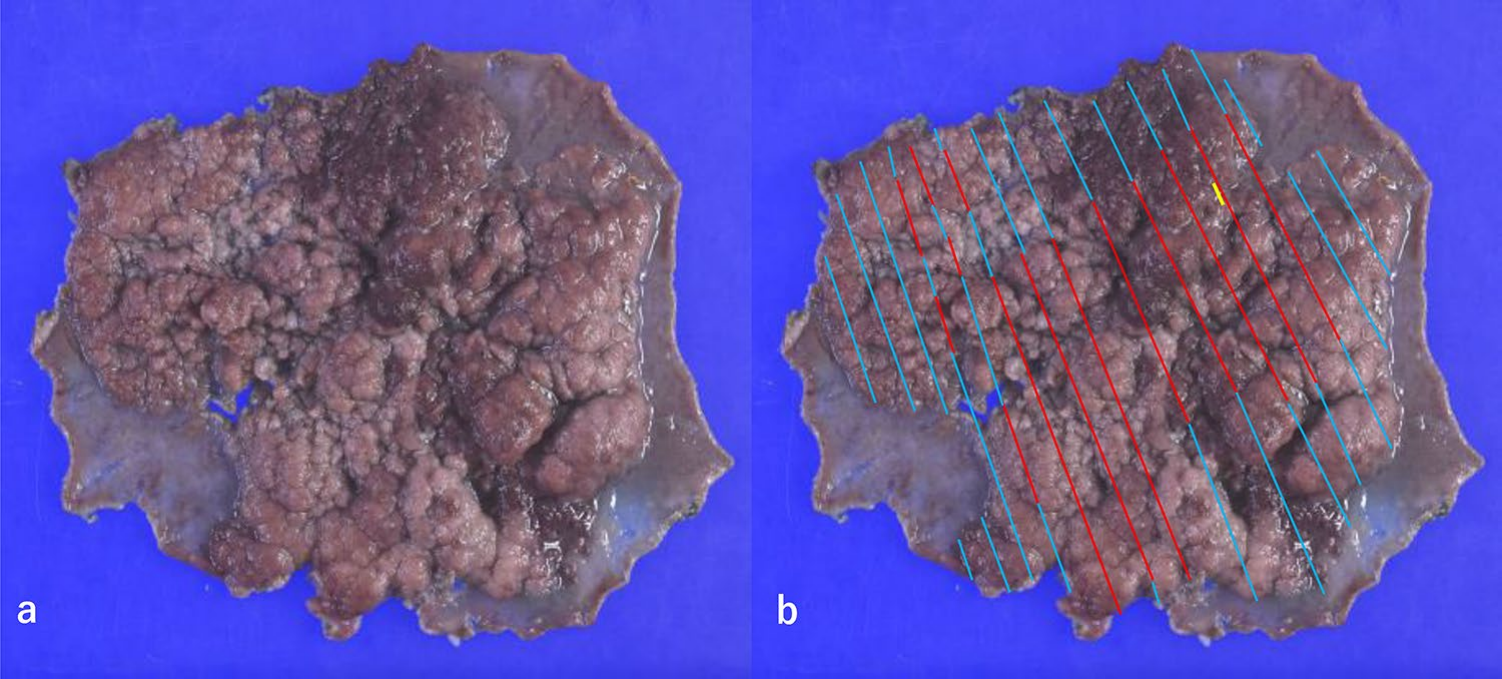
Pathology specimen of endoscopic submucosal dissection (ESD) reveals NEC component mixed with adenocarcinoma (**a**). Mapping of adenocarcinoma, adenoma, and NEC. Red lines indicate adenocarcinomas, blue lines indicate adenomas, and yellow lines indicate NEC (**b**). Where the lesions are almost exclusively adenocarcinoma, the NEC component is mixed in less than 3% of the lesions. NEC, neuroendocrine carcinoma

**Fig. 4:**
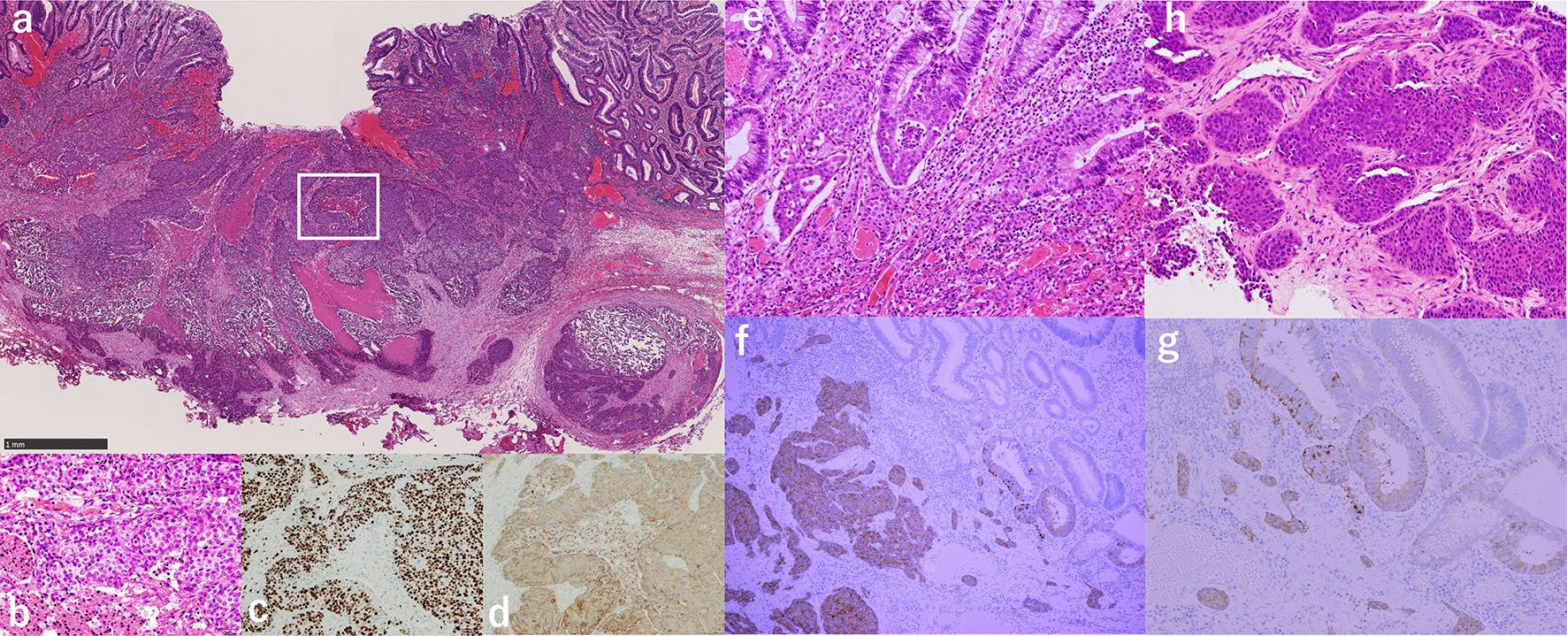
Histopathological findings of the specimen. **a** Neuroendocrine carcinoma in tubular adenocarcinoma (hematoxylin–eosin stain × 4). The area enclosed by the white square represents the NEC component. The white squares, magnified 200 times, are **b**, **c**, and **d**. **b** Neuroendocrine carcinoma (hematoxylin–eosin stain × 200). **c** Neuroendocrine carcinoma (MIB1 stain × 200). **d** Neuroendocrine carcinoma (Synaptophysin stain × 200). **e** Glandular epithelium, where NEC components are found to follow the shape of the glandular ducts. **f** Some of the adenocarcinomas bordering the NEC are synaptophysin positive (synaptophysin stain × 100). **g** A ×200 magnification of the enlarged portion of the adenocarcinoma (synaptophysin stain × 200). **h** Liver biopsy shows a histology similar to that of the NEC seen in the colon specimen

A 69-year-old woman was referred to our institution for treatment of a large flat-elevated rectal tumor. Colonoscopy (CS) revealed that the tumor involved a half-circumferential surface and covered the area above and below the anterior peritoneal reflection, extending to the anal margin (Fig. 1a). The whole lesion was soft and magnifying chromoendoscopy using crystal violet staining identified a type IV pit pattern (Fig. 1b). Thus, we diagnosed the lesion as an adenoma or intramucosal adenocarcinoma. Computed tomography (CT) showed no lymph node metastasis or obvious distant metastasis (Fig. 2a), and ESD was performed. Although intraoperative microperforation occurred, en bloc resection was successfully performed. The microperforation was treated conservatively, and CT was repeated after 1 week. The repeated CT showed a liver mass suspicious of metastasis (Fig. 2b) and Gd-EOB–DTPA-enhanced magnetic resonance imaging (MRI) showed multiple liver metastases (Fig. 2c, d). The resected specimen was 103 × 88 mm in size and contained an 88 × 82 mm tumor. Histopathological analysis showed tubular adenocarcinoma with an NEC component. The final pathological diagnosis was: rectal cancer, ESD.- type 0–IIa, 68 × 60 mm (carcinoma size), adenocarcinoma (tub1 > tub2) with neuroendocrine carcinoma component, pT1b at least (SM,3.8 mm), INFb, Ly1b (D2-40), V1b (EVG), Pn0, BD1, pHM0 (adenoma positive), pVM1. The NEC component was less than 3% of the total tumor, measuring about 5 mm (Fig. 3), and was found in the most advanced part of the infiltrate (T1b at least; SM3.8 mm) (Fig. 4a, b). The NEC component was positive for synaptophysin (Fig. 4c), negative for chromogranin A, very focally positive for CK5/6, and negative for p40. In addition, MIB1 labeling was 70% (Fig. 4d), necrosis was present, and the nuclear fission image was approximately 28/10 high power fields. NEC components were found to follow the shape of the glandular ducts (Fig. 4e). In addition, some of the adenocarcinomas bordering the NEC stained positive for synaptophysin (Fig. 4f, g). Liver metastasis was thought to be caused by the NEC component. Throughout the course of disease, a liver biopsy was performed, which confirmed that the liver metastasis was due to the NEC component (Fig. 4h). The patient then decided to proceed with chemotherapy, and irinotecan plus cisplatin was selected as the first line.

## Discussion

In this report, we encountered a rare case. Endoscopically, although the lesion appeared to be a normal ESD-eligible adenoma or adenocarcinoma, a very tiny fraction of the lesion (< 3%) contained an NEC component. However, the NEC component was found to be the most advanced part of the infiltrate, it rapidly led to multiple metastases.

Since MiNEN requires 30% or more of each of NET and non-NET components, this case is not strictly classified as MiNEN according to the 2019 WHO classification.

The characteristic endoscopic finding of gastrointestinal NET could be an analogous submucosal tumor-like elevation, accompanied by central depression and ulcer formation if it increases [6]. It has been reported that NEC and MiNEN often take the form of Borrman I or II advanced cancer [7]. In this case, although preoperative endoscopy showed large lesion area, the lesion was flat with inconspicuous thickness, and there was no evidence of deep invasion. Therefore, we considered this as a good indication for ESD and did not suspect that the NEC component was included.

Cases such as the present one, in which the NEC component was mixed with some of the adenocarcinoma, are relatively rare. Some reports have suggested that adenoma might progress through adenocarcinoma to NEC [8, 9]. In such cases, several genetic abnormalities are thought to occur for causing this transition from adenocarcinoma to endocrine carcinoma, one of which is the *CDKN2A* gene abnormality [6].

Recently, the concept of mixed epithelial endocrine neoplasms (MEENs) has been proposed, and this case is considered to fit this concept [10]. MiNEN arbitrarily sets the cutoff value for neuroendocrine component at 30%, based on a suggestion that tumor component of less than 30% could be insufficient to impact prognosis. However, recent findings have indicated that high-grade components, even if they represented less than 30% of tumor volume, may have a prognostic impact. The 2022 WHO classification of endocrine and neuroendocrine tumors states that diagnosis of non-gastrointestinal MiNEN is independent of quantity and depends on identification of two morphologically distinct components [11]. Regarding MiNEN in the digestive system, a cutoff value of 30% is tentatively maintained. However, it is suggested that future studies may change this classification. The MEEN concept does not set a cutoff value for each component and encompasses a wide range of heterogeneous tumors in diverse combinations ranging between high-grade, intermediate-grade, and low-grade tumors.

There were areas in the glandular epithelium, where NEC components were found to follow the shape of the glandular ducts. In addition, some of the adenocarcinomas bordering the NEC were positive for synaptophysin. These images suggest both the possibility of NEC invasion into the glandular ducts and NEC originating from them. Since the proportion of NEC component in the lesion was very small, the NEC was considered, in this case, to originate from an adenocarcinoma.

The point of reflection in this case is that the presence of liver metastasis could not be detected on preoperative CT. A retrospective observation showed one fairly obscure small faint shadow of approximately 7–8 mm. However, it was not confirmed by the radiologist or by us. Repeat CT 1 week after perforation showed that the shadow had increased in size to approximately 20 mm, with a clear outline, and was identified as liver metastasis. Although repeat CT also showed only one shadow, EOB–MRI showed a total of eight masses, five in the right lobe and three in the left lobe of the liver. Therefore, tumor progression was considered rapid. However, if the biopsy by ESD was not performed, it would have taken a long time to diagnose the adenocarcinoma as having a mixture of NEC components. From this point of view, the importance of en bloc resection by ESD was reaffirmed in this case.

We encountered a rare case of NEC mixed with adenocarcinoma component. Further accumulation of cases of lesions with mixed neuroendocrine and non-NETs is warranted.

## References

[1] Nagtegaal ID, Odze RD, Klimstra D (2020). The 2019 WHO classification of tumours of the digestive system. Histopathology.

[2] Ito T, Igarashi H, Nakamura K (2015). Epidemiological trends of pancreatic and gastrointestinal neuroendocrine tumors in Japan: a nationwide survey analysis. J Gastroenterol.

[3] Park CH, Cheon JH, Kim JO (2011). Criteria for decision making after endoscopic resection of well-differentiated rectal carcinoids with regard to potential lymphatic spread. Endoscopy.

[4] Hotta K, Shimoda T, Nakanishi Y (2006). Usefulness of Ki-67 for predicting the metastatic potential of rectal carcinoids. Pathol Int.

[5] Garcia-Carbonero R, Sorbye H, Baudin E (2016). ENETS consensus guidelines for high-grade gastroenteropancreatic neuroendocrine tumors and neuroendocrine carcinomas. Neuroendocrinology.

[6] Kim BN, Sohn DK, Hong CW (2008). Atypical endoscopic features can be associated with metastasis in rectal carcinoid tumors. Surg Endosc.

[7] Tanaka T, Kaneko M, Nozawa H (2017). Diagnosis, assessment, and therapeutic strategy for colorectal mixed adenoneuroendocrine carcinoma. Neuroendocrinology.

[8] de Mestier L, Cros J, Neuzillet C (2017). Digestive system mixed neuroendocrine-non-neuroendocrine neoplasms. Neuroendocrinology.

[9] Li Y, Yau A, Schaeffer D (2011). Colorectal glandular-neuroendocrine mixed tumor: pathologic spectrum and clinical implications. Am J Surg Pathol.

[10] Kanthan R, Tharmaradinam S, Asif T (2020). Mixed epithelial endocrine neoplasms of the colon and rectum - an evolution over time: a systematic review. World J Gastroenterol.

[11] Rindi G, Mete O, Uccella S (2022). Overview of the 2022 WHO classification of neuroendocrine neoplasms. Endocr Pathol.

